# Real-Time Needle Force Modeling for VR-Based Renal Biopsy Training with Respiratory Motion Using Direct Clinical Data

**DOI:** 10.1155/2019/9756842

**Published:** 2019-06-25

**Authors:** Feiyan Li, Yonghang Tai, Qiong Li, Jun Peng, Xiaoqiao Huang, Zaiqing Chen, Junsheng Shi

**Affiliations:** ^1^Yunnan Key Laboratory of Opto-electronic Information Technology, Yunnan Normal University, Kunming, China; ^2^Department of Urology Surgery, Yunnan First People's Hospital, Kunming, China

## Abstract

Realistic tool-tissue interactive modeling has been recognized as an essential requirement in the training of virtual surgery. A virtual basic surgical training framework integrated with real-time force rendering has been recognized as one of the most immersive implementations in medical education. Yet, compared to the original intraoperative data, there has always been an argument that these data are represented by lower fidelity in virtual surgical training. In this paper, a dynamic biomechanics experimental framework is designed to achieve a highly immersive haptic sensation during the biopsy therapy with human respiratory motion; it is the first time to introduce the idea of periodic extension idea into the dynamic percutaneous force modeling. Clinical evaluation is conducted and performed in the Yunnan First People's Hospital, which not only demonstrated a higher fitting degree (AVG: 99.36%) with the intraoperation data than previous algorithms (AVG: 87.83%, 72.07%, and 66.70%) but also shows a universal fitting range with multilayer tissue. 27 urologists comprising 18 novices and 9 professors were invited to the VR-based training evaluation based on the proposed haptic rendering solution. Subjective and objective results demonstrated higher performance than the existing benchmark training simulator. Combining these in a systematic approach, tuned with specific fidelity requirements, haptically enabled medical simulation systems would be able to provide a more immersive and effective training environment.

## 1. Introduction

Minimally invasive surgery (MIS) is prevalent in the medical field due to the characteristics of efficiency, less bleeding, and faster recovery [1]. Biopsy therapy, one of the commonest MIS, is a kind of needle insertion medical procedure, assisted by a real-time medical image (ultrasound/CT), which navigates the needle percutaneously inserted through the human trunk to reach the lesion target. Since the needle insertion goes through different tissue layers, such as the skin, fat, and muscle, to finally reach an internal organ [2, 3], it is hard to handle the puncture position angle and force during the operation; consequently, MIS has strict requirements for surgeons: they must undergo a long training period to gain surgical experience, which means the curve of surgical learning on this minimally invasive surgery is extremely steep. Inexperienced surgeons and interns usually use animals, artificial models, and cadavers for surgical training, but since the material characteristics of artificial models are quite different from real human tissues, they cannot achieve the effect of training because of the lack of reality. Apart from that, the source of cadavers is limited, and from the perspective of the concept of protecting animals, animals used for surgical training are also subject to ethical restrictions. For inexperienced medical personnel, multiple surgical training is inevitable, especially for difficult operations, such as the lung, liver and other related operations. Therefore, it is important to provide a good surgical training platform for the inexperienced medical personnel and interns. Surgical procedure simulations and robot-assisted surgery provide a good training platform for MIS training. Virtual reality (VR) emerged as an essential technology of medical education applications in recent years; benefiting from these cutting-edge facilities, surgical procedure simulations and robot-assisted surgery have become increasingly immersive and reliable by adding visual and haptic feedback rendering [4–6]. Nevertheless, the challenge of accurate and realistic haptic feedback cannot be overlooked, due to the high-frequency update requirement (1000 Hz). The big problem is the contradiction between accuracy and real time; to improve the accuracy of the system, it is necessary to make appropriate sacrifices in real time; many existing force calculation models based on either artificial or animal-based experimental data cannot truly reflect the mechanical behavior response of soft tissues, thus failing to meet the force feedback authenticity requirements [7, 8]. In this paper, we proposed an immersive dynamic haptic sensation model during biopsy therapy with human respiratory motion evaluated by intraoperative clinical data. The major contribution of this work is the higher fitting degree force modeling based on real-time clinical data. In addition, a dynamic biomechanics experimental framework is also designed to provide higher specification and an immersion haptic rendering scenario. Last but not least, the proposed VR-based biopsy training evaluation simulator complements the training immersion and minimizes the gap between virtual and reality. To the best of the authors' knowledge, it is innovative to integrate intraoperative surgical data into dynamic haptically enabled real-time VR medical training simulation.

The structure and content of this article are organized as follows: we briefly make an introduction on biopsy surgery and the challenges of VR-based medical training in the above-mentioned part; after that, we review the previous related work on needle insertion biomechanics modeling. Thirdly, the biomechanics data-based force model, with clinical and VR training evaluation experiments, is designed in Methodology. Fourthly, the experimental fitting and evaluation results were demonstrated and discussed. Finally, we summarize the results and the potential contributions this paper makes.

## 2. Related Work

Currently, lots of researchers have studied the needle insertion and proposed many mechanical models based on the experimental data. The related work can be classified into two species according to data acquisition: in vivo and in vitro. In the first species, Brouwer et al. [9] and Brown et al. [10] carried out the experiment from in vivo soft tissue to analyze the properties of soft tissue and describe the insertion force profile. For force modeling, Maurin et al. [11] performed a series of experiments; they compared the maximum force of different organs during insertion in vivo and even gave a conclusion about the difference between manual and robotic. Barbé et al. [12] found a method to estimate online the needle forces with a low velocity of the needle tip and analyzed the modeling and the interaction between the needle and tissue during in vivo experiments. Moreover, researchers also developed a simulation system for insertion and measured the biomechanical properties of in vivo soft tissue. Kobayashi et al. [13] studied an integrated robot-assisted system with ultrasound-guided and simulation soft tissue deformation using in vivo experimental data on the porcine liver; the verification showed that the insertion simulation can locate the needle tip into the target accurately. Menciassi et al. [14] have proposed a novel instrument and method to measure in vivo biomechanical properties of tissue. In addition, Han et al. [15] developed an ultrasound indentation system to efficiently measure soft tissue mechanical properties in vivo. In the second species, many researchers focus on modeling insertion force; Simone and Okamura [16] took an interest on in vitro experiments on bovine livers using the Johns Hopkins University Steady Hand Robot for percutaneous therapy, and the needle insertion forces were modeled based on the properties of soft tissue. The insertion forces were divided into three components: capsule stiffness, friction, and cutting. Okamura et al. [17] also presented a model for insertion force and experiment for acquiring data from ex vivo liver tissue; the insertion process has two phases: prepuncture and postpuncture, where the stiffness force belonged to the former and friction force and cutting force are the latter. The experiment was performed to describe the effects of friction force and needle geometry during the robotic needle insertion into tissue. Moreover, the experiment setup was constructed for friction tests under the CT scanning imaging. Some proposed a new puncture object to replace the animal soft tissue [17]. Ng et al. [18] found an appropriate method to model the interaction forces during needle insertion into porcine back tissue and simulated multilayer gelatin; the porcine sample was performed in vitro to collect force data. The insertion force with simulated multilayer gelatin compared with porcine sample shows that the simulated multilayer gelatin can simulate the cutting force. Thus, its insertion force resembles the actual porcine experiment and provides a suitable alternative medium for design and training needle insertion. And other people studied the efficiency factor of insertion forces. Jiang et al. [19] introduced the mechanism and influencing factors of the interaction between puncture needle and soft tissue in detail; the result showed that the effect of force has lots of factors, for example, needle geometry and soft tissue type. Gessert et al. proposed a new method for needle tip force estimation by using OCT fiber imaging, and an ex vivo experiment with a human prostate was tested [20]. Apart from that, lots of researchers focus on realizing the haptic feedback during needle insertion by using some of the existing commercial force feedback equipment, such as the PHANTOM haptic device [21]. In 2017, Xue et al. presented a teleoperated needle insertion system with haptic feedback; they designed a novel force control framework to match the force signals, and the relationship between motor current and feedback force is modeled [22]. In 2018, Yang et al. summarized the key components surrounding the force feedback and control during robot-assisted needle insertion; this review involved force modeling, identification, and feedback control of robot-assisted needle insertion [23].

According to the reviewed literature above, it can be summarized that two major flaws will appear when integrating these models with the biopsy simulation: most of the experimental data recording was performed under the static condition, without the respiratory motion; however, the breath action drives the human viscera up and down throughout the biopsy procedures. Moreover, there is no doubt that experimental force data from in vivo is more reliable than in vitro, but are these animal experimental data-based force models suited for the haptic rendering in the VR-based training simulator of human biopsy surgery? With respect to these points, we conducted the solutions with a dynamic biomechanical experiment platform and evaluated with clinical data in the following parts.

## 3. Methodology

### 3.1. Biomechanics Data-Based Force Model

#### 3.1.1. Biomechanics Test Platform

Respiratory motion is an unavoidable influencing factor of the needle insertion precision during the biopsy surgery; nevertheless, most of the researchers focus the study on the static puncture in vitro. Force is recorded in the ideal without-breath condition, regardless of the movement of the organ by the respiration during the operation. To resolve this issue, this study designed a novel biomechanical platform to record the dynamic biopsy force with respiratory motion. According to Weiss et al.'s research [24], the primary motion of human organs' 3D centroid is the head-foot direction movement; based on the 4D-CT data, the tissue has simple motion ranges [25–27] and the parameters we chose in this work are demonstrated in Table 1.

**Figure 1:**
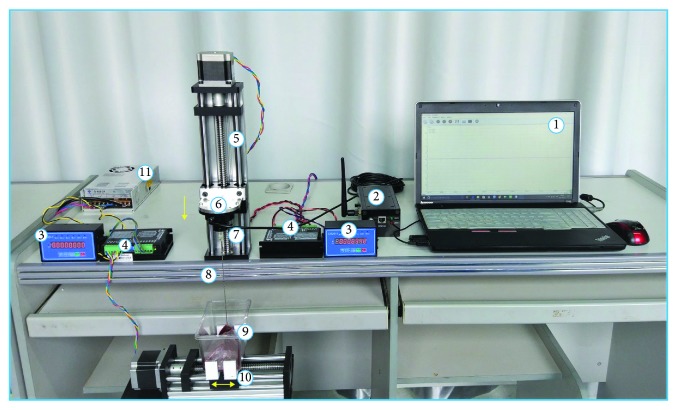
The entire simulation system of the biomechanics experiment. 1: acquisition software; 2: multichannel data conversion card; 3: servomotor controller; 4: servomotor actuator; 5: linear guideway (vertical); 6: connector; 7: force sensor (ATI Nano17); 8: surgical trocar needle; 9: biopsy sample; 10: linear guideway (horizontal); 11: power supply.

The biomechanics test platform mainly consists of the surgical instrument, force sensor acquisition system, stepper motor drive system, container for soft tissue, and computer, which are detailed and demonstrated in Figure 1. The biomechanics data is collected from 5 different part tissues of porcine (in Table 1), which are bought from the butcher market within 24 hours. The experiment temperature is 19 ± 0.5°C, humidity is 25%, and the biopsy needle parameter is 18G trocar with a 1.06 mm inner diameter and 15 cm long. We select ATI Nano17 as the force sensor; it is installed on the handle of the trocar to collect the information of the insertion force in real time; the precision is 0.01 N. The stepper motor is mainly composed of a slider, subdivision, and controller, the maximum motion range is 200 mm, motion accuracy is 0.16 *μ*m, and the velocity can be programmed to for control, with the controllable range of 1 mm/s-30 mm/s. There are two stepper motor drive systems: one is used to control the vertical velocity of the biopsy needle insertion and the other one is used to simulate the human respiratory motion to achieve dynamic puncture. The literature suggested that the velocity of biopsy needle insertion is usually in the range of 0.1 mm/s~10 mm/s [19]; therefore, we set the needle insertion with the constant velocity of 3 mm/s in this study. The size of the container is 12 mm × 7 mm × 7 mm, and the sampling frequency of the force sensor is 1000 Hz. Apart from that, the experimental platform must be placed on the horizontal table to avoid the chattering phenomenon during needle insertion; the needle tip and the soft tissue surface are in a vertical state and have a distance, which ensures the trocar can transform from acceleration to the uniform motion. The whole experimental setup is shown in Figure 1. In order to reduce the experimental error, each kind of tissue experiment was performed on three different regions and then the average value is calculated; only the average value is determined as the analysis data for the force modeling.

#### 3.1.2. Insertion Force Modeling

The mechanical properties of soft tissues are complex and usually exhibit characteristics such as nonlinearity and anisotropy [28]. The study of their mechanical properties is mainly to analyze the stress-strain relationship, but there is no clear mechanical model to define it strictly because the internal structure of the soft tissue is complex; most of them are based on the properties of soft tissue and experimental data to analyze the mechanics modeling. Simone and Okamura [16] used a nonlinear spring model as a model for the stiffness and a modified Karnopp model as a model for the friction; the cutting was obtained by subtracting friction from the whole forces; it can be considered as a constant for a given material. Okamura et al. [17] found that it was best to fit the data by a second-order polynomial for prepuncture. And Maurin et al. [11] analyzed the experimental data using an exponential function of the depth in each puncture phase. Some scholars assume that the soft tissue has a linear characteristic and propose a linear mechanical model, but this does not meet the objective requirements and the actual needs.

As far as we know, the needle puncture procedures are continuity, uncertainty, and independence, which may be hard to describe with the existing model. Therefore, it is necessary to find a mechanical model that can be applied to both the puncture process analysis and the general application. As a powerful function, Fourier is usually used in various fields; consequently, Fourier is applied to insertion force modeling in this paper. In order to incorporate the Fourier model into the mechanical modeling of percutaneous biopsy, we introduced the periodic extension idea to convert the nonperiodic puncture force into a periodic function while extending the function definition domain to the entire real field. Because the real-time puncture force is centered on the low-frequency part, according to Fourier definition, each periodic function can be decomposed of numbers of trigonometric periodic functions, which is the Fourier series. The equation is as follows:

(1) $$\begin{array}{c} y\left(\text{x}\right)=\alpha_{n}\cos\left(n\omega x\right)+\beta_{n}\sin\left(n\omega x\right). \end{array}$$

Because of the Euler formula: *e*^*ix*^ = cos*x* + *i*sin*x*, we transformed the trigonometric function into an exponential form:

(2) $$\begin{array}{c} y\left(x\right)=\overset{\infty }{\underset{n=-\infty }{\sum }}m_{n}e^{in\omega x}, \end{array}$$

(3) $$\begin{array}{c} y\left(x\right)=\alpha_{0}+\overset{\infty }{\underset{n=1}{\sum }}\left(\alpha_{n}\cos\left(n\omega x\right)+\beta_{n}\sin\left(n\omega x\right)\right)\;, \end{array}$$

(4) $$\begin{array}{c} \alpha_{0}=\frac{2}{T}\int_{0}^{T}y\left(x\right)dx, \end{array}$$

(5) $$\begin{array}{c} \alpha_{n}=\frac{2}{T}\int_{0}^{T}y\left(x\right)\cos\left(n\omega x\right)dx,n=1,2,\cdots \infty , \end{array}$$

(6) $$\begin{array}{c} \beta_{n}=\frac{2}{T}\int_{0}^{T}y\left(x\right)\sin\left(n\omega x\right)dx,n=1,2,\cdots \infty , \end{array}$$

(7) $$\begin{array}{c} m_{n}=\frac{1}{T}\int_{0}^{T}y\left(x\right)e^{-in\omega x}dx,n=0,\pm 1,\pm 2,\cdots \infty . \end{array}$$

Based on the above formula, the superposition coefficient of a function expression with a known periodic function can be obtained by integration. In our simulation, since the force model is unclear, we employed the function approach method to fit the experimental data:

(8) $$\begin{array}{c} \underset{N\to \infty }{\lim}\int_{0}^{T}{\left|y\left(x\right)-\left[\alpha_{0}+\overset{\infty }{\underset{n=1}{\sum }}\left(\alpha_{n}\cos\left(n\omega x\right)+\beta_{n}\sin\left(n\omega x\right)\right)\right]\right|}^{2}dx=0. \end{array}$$

According to this, we introduced finite triangular periodic functions approaching the puncture force function *y*(*x*) to describe the final model as

(9) $$\begin{array}{c} y\left(x\right)=\alpha_{0}+\overset{N}{\underset{n=1}{\sum }}\left(\alpha_{n}\cos\left(n\omega x\right)+\beta_{n}\sin\left(n\omega x\right)\right). \end{array}$$

**Figure 2:**
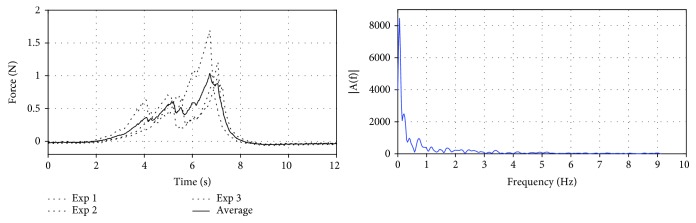
The insertion force of the kidney and corresponding frequency spectrogram.

*α*_*n*_ and *β*_*n*_ are the Fourier coefficients, and *N* means the term number of the Fourier series. In Figure 2, the force model is analyzed through frequency spectrogram to determine the Fourier series *N*, which is set from 1 to 8.

### 3.2. Clinical Evaluation Experiment Design

#### 3.2.1. Clinical Data Collection Platform Design

**Figure 3:**
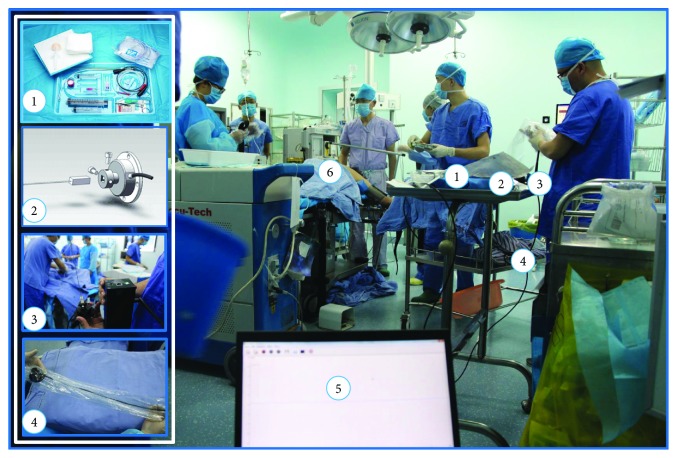
Clinical trial setup for the multichannel intraoperative data collection. Component details are as follows: 1: biopsy surgical instrument; 2: force sensor (ATI Nano17) +3D print connector; 3: line puncture device integration; 4: data conversion; 5: analysis software; 6: patient.

We chose the renal biopsy surgery as the clinical data validation procedure which is an invasive procedure to obtain a small piece of tumor sample from the renal tissue for pathologic examination. Clinical data collection trials are set up in the Division of Urology in Yunnan First People's Hospital, Kunming, Yunnan, China. The needle insertion usually can be divided into prepuncture and postpuncture; before insertion, the patient lies in the supine position, then the operator injects local anesthesia into the skin, through the subcutaneous tissue and down to around the kidney. After the preliminary work is prepared, a biopsy is performed by the operator with the aid of real-time medical imaging for guidance. It provides critical information for clinicians to determine the condition, treatment prescription, and postoperative estimation. In these procedures, what it is desired is more accuracy of insertion, reduced operation time, and less damage to surrounding tissue during the insertion. Because of one of the coauthors who is also a professional surgeon in the hospital, all clinical and data collection procedures referring to the patients and the surgery are anonymous and nonidentifiable. Clinical procedures are planned and conducted by professional urologists in the hospital and in accordance with associated safety and ethical approvals. The clinical data adopted in this paper was provided to the authors through a public link to a database on haptic force feedback (http://civ.ynnu.edu.cn/ChineseShow.aspx?ID=1).  Figure 3 shows the clinical system setup for clinical data collection and the detailed system components in the surgical room implementation.

#### 3.2.2. Experiment Setup

**Figure 4:**
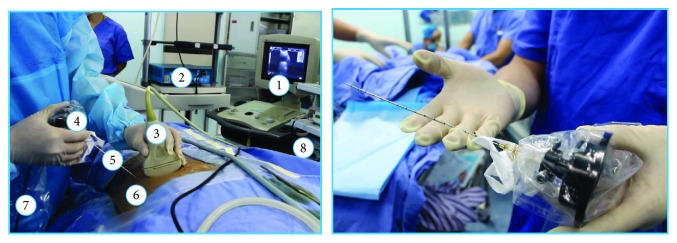
Percutaneous intraoperative force recording instrument includes a 6 DoF force sensor, a biopsy needle, and a customized 3D printed connector.

The actual percutaneous therapy involves a urologist holding the percutaneous instrument to conduct the percutaneous surgery while real-time force data is recorded. The procedure involves a force acquisition component (ATI Nano17) and a guide image recording component (ultrasound), as well as an audio recording component. The force acquisition component is implemented based on a standard trocar needle (18G COOK trocar needle), which is a flexible surgical instrument, consists of a needle core and needle sheath which is demonstrated in Figure 4, and is specifically for such procedures. A force sensor, a data collector, and a power supply were also integrated with the trocar needle, as shown in Figure 4. The force sensor is an ATI Nano17. It connects to the rear end of the trocar needle through a customized 3D print connector.

**Figure 5:**
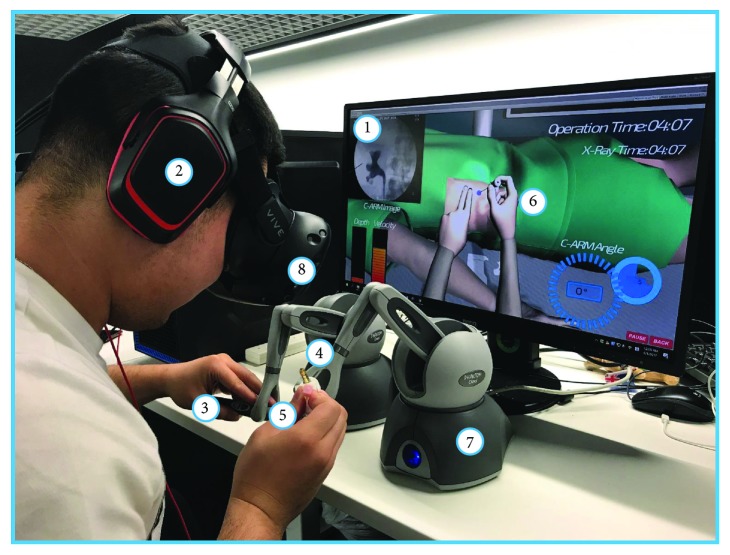
The immersive virtual training platform with an i7 6700 (3.4 GHz) CPU workstation, 8 GB memory, and 1070 NVIDIA Graphics GPU. Two PHANTOM Omni reproduce the dual-hand force rendering, HTC VIVE provides the 3D visual rendering with head tracking, and a Beat Solo 2 wireless headphone provides the real-time surgical audio. Component details corresponding with the operation room are demonstrated in Table 2.

As the CT images used for visual reconstruction could be obtained before the operation, only the guided image recording is required in real time. Ultrasound images were recorded with a screen capture software as a guiding image. Each frame was recorded for the guide image in the proposed training environments. The recorded images are cropped to the preoperation part as the training interface needs to be overlaid with only a shadow's projection of the needle. On the audio recording part, we adopted a Sony PCM-D100 high-resolution recorder to capture the working audio of machines inside a theatre. We then replay these as the background audio in our training system.  Table 2 shows the biopsy surgical components in Figure 4 and the corresponding components in a virtual simulator in Figure 5.

### 3.3. VR-Based Training Evaluation Simulator Design

#### 3.3.1. Visual and Haptic Rendering Pipeline

**Figure 6:**
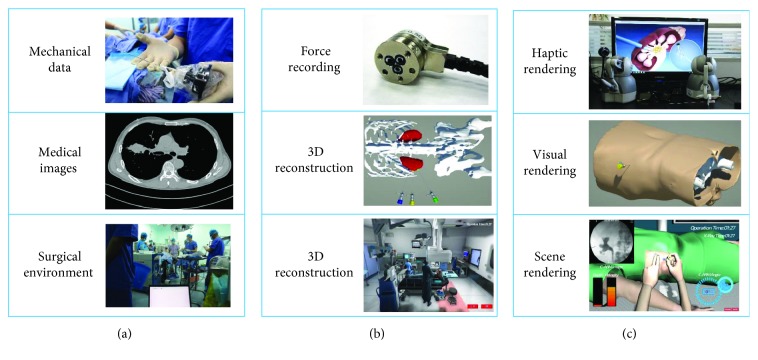
The implementation of the medical training platform with direct intraoperative data, which includes the multichannel data recording in the operation room (a), data processing and reproduction (b), and final virtual medical training environment reconstruction (c).

In the proposed training framework, the urologist guides the real-time C-ARM or ultrasound image navigation to locate the minimal invasive position for needle insertion. To provide an immersive haptic rendering of such dual-hand operation, we implemented both ultrasound force and needle insertion force within one haptic rendering loop. The first line in Figure 6 demonstrates the proposed two different haptic rendering schematic, which could potentially be used in many other image-guided medical simulations. Visual rendering is responsible for visual clue setup according to an anonymous CT dataset provided by the Yunnan First People's Hospital, Kunming, China. The dataset has a dimension of 512 × 512 × 3172 and 0.51 × 0.51 × 0.50 mm accuracy, as demonstrated in the second line of Figure 6. The CT dataset (DICOM format) was first imported for ROI extraction using segmentation functions such as threshold and region growing. Afterwards, 3 urologists were invited from Yunnan First People's Hospital to validate the autosegmentation result and perform manual correction where necessary, as demonstrated in the second line of Figure 6. Finally, we employed the marching cube algorithm to reconstruct the dataset into a 3D mesh model, with redundant mesh cleaning and Laplacian smoothing as intermedium steps. Finally, we reconstructed the whole surgical environments in MAYA and added the background audio inside into the scene rendering shown in the third line of Figure 6. Two PHANTOM Omni reproduce the dual-hand force rendering, HTC VIVE provides the 3D visual rendering with head tracking, and a Beat Solo 2 wireless headphone provides the real-time surgical audio.

#### 3.3.2. System Evaluation Design

18 novice and 9 expert urologists are invited to assess the virtual reality training platform. Before the training session starts, a didactic introduction of system operation is provided to both the expert and novice groups. Furthermore, a customized-built Global Rating Scale (GRS) questionnaire is introduced for the subjective evaluation [29–31]. The time interval of an objective test is 30 min between the pretest and posttest groups of the novices. The time interval between the training sessions is 24 hours. After finishing the experiments, all 27 trainees' performance was recorded by the subjective questionnaires to evaluate our biopsy therapy training system with respiratory motion. The evaluation system procedures are demonstrated in Figure 5.

**Figure 7:**
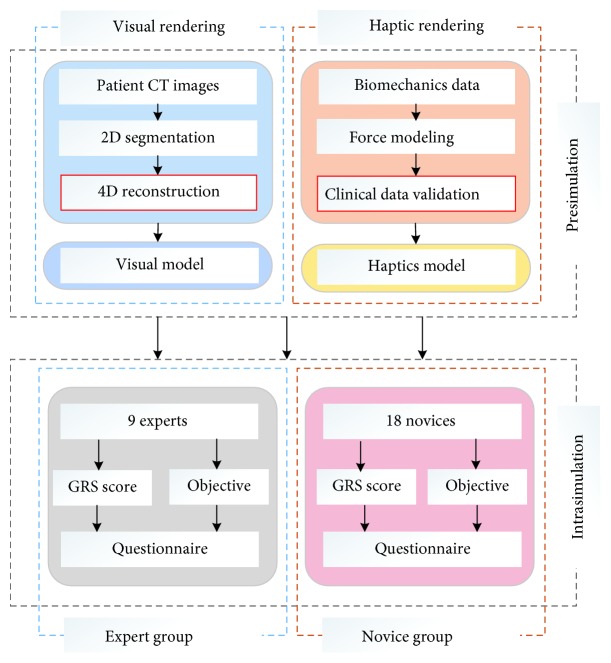
The evaluation system procedure design underwent our immersive virtual percutaneous therapy simulator.

Comparisons between groups (novice–expert) were analyzed using the Mann–Whitney *U* test (continuous variables) or the chi-square test (categorical variables) of the stored data in our system [6, 32]. Pretraining and posttraining data in the 2 groups were compared using paired *t*-tests. Spearman rank order correlations were calculated to determine correlations between objective simulator-derived scores and the GRS results. *P* value < 0.05 was considered statistically significant. Analyses were performed using the software package IBM SPSS Statistics, version 20.0 [33, 34]. The evaluation system procedure design underwent our immersive virtual percutaneous therapy simulator as shown in Figure 7.

## 4. Results

### 4.1. Results of Biomechanics Force Modeling

**Figure 8:**
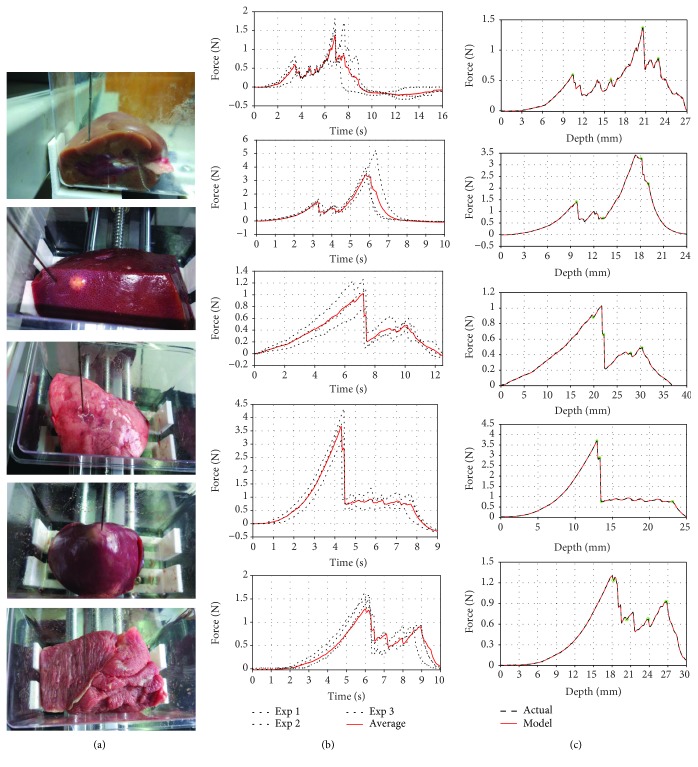
The biomechanics force data fitting results: tissue sample (a), initial data and average (b), and mean-value force modeling with our proposed force model (c).

After the experiments, the data were analyzed by MATLAB 2016 software. The nonlinear least squares were utilized to fit the insertion force by using the Fourier model proposed in Section 3.1.2. To avoid coincidence, three different regions of the same individual are performed for comparing the tendency of insertion force; finally, the average value was calculated and then the insertion force was modeled except the retraction phase. The relationship curve between the insertion force and the time is shown in Figure 8; as can be seen from the figure, the tendency of the insertion force has broad resemblance but the position of the peaks and valleys of the insertion force has a little deviation due to the anisotropy and inhomogeneity of the internal tissue of the soft tissue. Therefore, for the same object, there is a tiny difference in the data curve for different regions; mechanical modeling can be used in the same model [35].

A detailed piecewise fitting degree of five kinds of tissues is demonstrated in Table 3; parameters of tissue movement during the respiration are set based on Table 1.

### 4.2. Evaluation Results of the Haptic Model by Clinical Data

**Figure 9:**
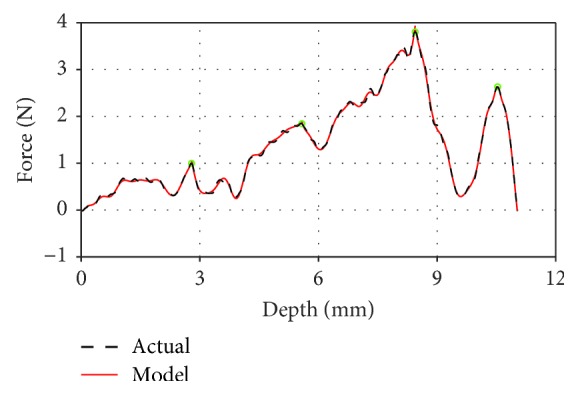
The fitting result of intraoperative data using our force model.

To verify the modeling method, the clinical trial setup is conducted in the Urology Department of the Yunnan First People's Hospital in Kunming; all the procedures related to the patients and the operation are designed and constructed by the professional surgeons according to the safety and ethical rules. Based on the result in Figure 9 and Table 4, the proposed dynamic model in this study demonstrated a high fitting performance with the interoperated data in a real operation.

### 4.3. Evaluation Results of the VR-Based Training Simulator

Each expert participant could perform the puncture attempts on the model and successfully hit the provided tumor in the calyx. The survey showed that 7 participants (77.8%) would use our framework to train novices and 8 urologists (88.9%) considered the kidney model as an accurate anatomic representation with only 1 participant (11.1%) who did not agree with this statement. 6 participants (66.7%) considered the depicted the graphic simulation is as real as the real surgical scene. The other 3 were neutral about this statement. 5 (55.5%) participants thought the system had a high performance of haptic feedback; 2 (22.2%) of them were neutral. 8 attendants (88.9%) considered the X-ray-simulated image as an accurate representation of a real fluoroscopic image, and all of them (100%) felt that avoiding radiation was important for training; the detailed score of GRS and the objective evaluation on the VR-based training simulator are shown in Table 5.

For the system subjective questionnaire evaluation result, novices' appraisement demonstrated significantly higher than experts' in total performance. Details of the subjective evaluation of the VR-based training simulator are shown in Table 6.

## 5. Discussion

As medical surgery, percutaneous operation is often utilized for cancer treatment, and cytopathological examination, as the cancer treatment method, such as percutaneous ethanol injection therapy (PEIT) and radiofrequency ablation (RFA) is performed for liver cancer; both of them require the needle insertion into a specific part of the diseased area as precise as possible [13]. For cytopathological examination, with the navigation of intraoperative images, residents usually rely on their clinical experience to manipulate the operation to extract the lesion sample. Both are widely used in the invasive procedure. Like Gordon et al. [35] based on the MATLAB program, the data was segmented into piecewise regions and modeled. In this work of the insertion force model, the Fourier series N was adjusted according to the different regional characteristics, and then the model parameter was estimated by the algorithm. We have some information from the fitting curve, the Fourier model can describe the experimental data well, and it has a good coincidence with the experimental data, especially the fluctuation phenomenon in the process of needle insertion. The mechanical model proposed in this paper has a good effect on the insertion force. In addition, a series of needle insertion experiments were performed to verify the applicability of the Fourier model. Next, needle insertion was performed on four different tissues: the liver, lung, heart, and pork. As with kidney insertion, each group experiment tested three different regions and calculated the average value, and each type of experimental data was analyzed and modeled using the Fourier model presented in this paper. The following are the result of data acquisition and modeling for each tissue type. From the above modeling results, the Fourier model has a high degree of fitting for insertion force, it is not accidental, and it is also applicable to each tissue type. And it is further illustrated that the Fourier model has a generalization. Moreover, there is obvious information according to the above experimental data of four tissue types:

- For different tissue types, the stiffness force and the force distribution are different
- For the same tissue type, the insertion force curves vary from region to region, but the tendency of the force curve is similar; only the force peaks and slopes have a little diversity
- The complexity of the organizational structure distribution is known from the shape of the mechanical curve by observing the fluctuation of the force curve during needle insertion. The distribution of the internal structure was simple when the fluctuation was calm, but vice versa

**Figure 10:**
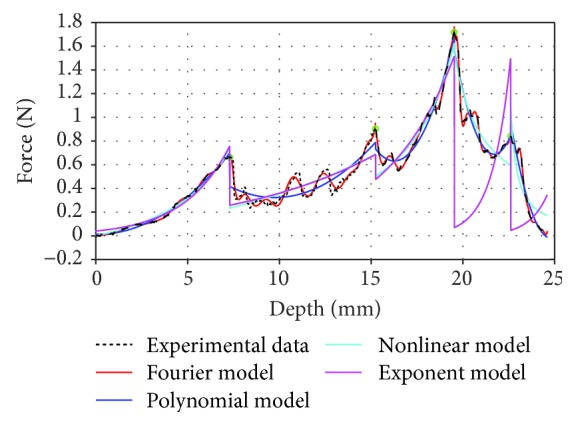
Comparison with the existing fitting algorithms of experimental data (left kidney).

In this paper, the experimental data were modeled using the above classical methods and the Fourier model proposed by us. As shown in Figure 10, it is obviously seen that the Fourier model has a better coincidence with the experimental data than the other models, especially when the insertion force fluctuates; the polynomial model and the nonlinear model have difficulty in describing the tendency of insertion force.  Table 7 shows the accuracy of each model to fit the insertion phase; it can be also seen from Table 7 that the Fourier model shows a high performance on the experimental data; the average of the fitting precision is 0.9855. Consequently, in this paper, we employed the Fourier model to analyze the whole needle insertion phase.

**Figure 11:**
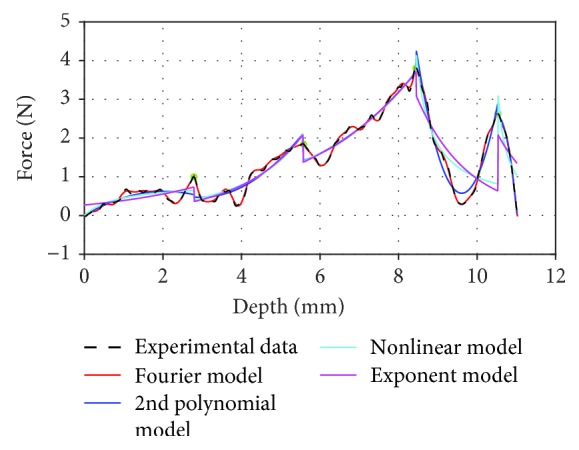
Comparison with the former fitting algorithms of clinical data (renal biopsy).

From Figure 11 and Table 8, we can see that the model this paper proposed can effectively simulate the interoperated data in a real operation. In addition, the experimental data of the kidney was utilized to compare with the intraoperative data; we find from the similarity from Figure 8 that there is a range of fluctuations in the force during needle insertion whether it is a manual insertion or a robot hand insertion; this phenomenon could have been contributed to by the soft tissue's material properties. But there is a difference between the intraoperative data of the manual control and the experimental data of the robot, in which the insertion force of the manual control is greater than the insertion force of the robot. Although the model of the insertion force we proposed shows great work in fitting experimental data and clinical data, what one should pay more attention to is the discontinuity and unsmoothness between nodes in the piecewise modeling; it is a deficiency of the Fourier model and must be modified. In the past years, the mechanical properties of biological soft tissues were studied through mechanical experiments. In recent years, research trends use theoretical models to analyze and fit experimental results. That is to say, a mathematical expression is used to describe the mechanical behavior of soft tissue by combining the theoretical model with experimental data. For simulating the puncture biopsy accurately, the next work is going to find a mechanical model considering the viscoelasticity and hyperelasticity of soft tissue. Moreover, collecting and enriching the database of soft tissue systematically will be an essential step in the future for the establishment of big data medical analysis.

To sum up, the principal contribution of this paper is to implement and verify the modeling method, by obtaining the clinical data of the needle puncture and comparing with the experimental data of a dynamic framework, which illustrated that the dynamic insertion model can effectively simulate the real operation.

**Figure 12:**
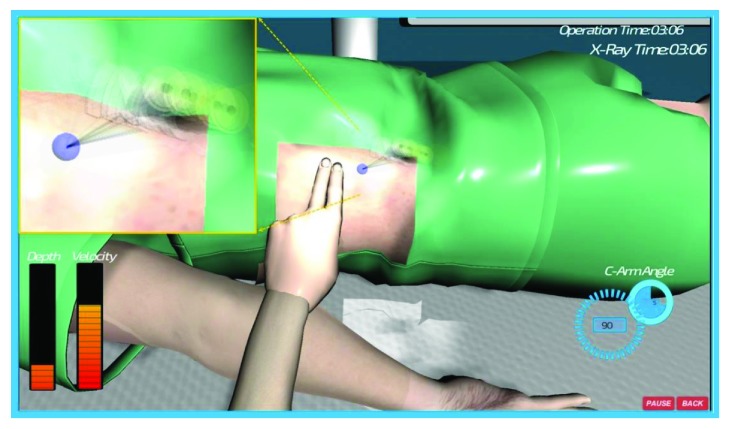
Real-time biopsy therapy VR training simulator with respiratory motion (left kidney).

For the system evaluation result, the face and content appraisal median value was 4 (1-5) by 9 experts, which is the same score as the existing percutaneous biopsy surgical training platform. Experts' performance is significantly higher than novices' in total performance. Posttest values of novice group after a few training sessions have shown significant improvement with respect to pretest GRS and objective scores. In all, Table 6 demonstrates that the proposed simulation platform's evaluation showed higher performance than the existing benchmark training platform. Simulation complexity and graphics on visualization showed a higher score with the novice students; this can be explained by the fact that the real ultrasound images are integrated into the operation face which provides an accurate visual indication for trainees. On the other hand, the training tools and assessment tool displayed a better performance with the novice; this result may have arisen by the fact that the needle simulator in the current version utilized the real surgical needle integrated with a low-fidelity stylus of the haptic device of the simulator, which may be confusing for the experts; moreover, the missing foot pedal fluoroscopy controls may have also led to the score deduction. Most of the experts still had doubts about considering it as an assessment tool and would prefer further validation studies to reach a conclusion. Besides the subjective validation on the face and content, more objective evaluations also require construct validity. To our knowledge, this study is the first time to validate a VR-based biopsy simulator by utilizing dynamic original clinical haptics, which provided a high-fidelity simulation of the biopsy construct validity in Table 5. Experts had significantly shorter resection times than novices. In addition, our simulator demonstrated a higher score in the “identify anatomy” and “overall performance.” The rib injury number in our simulator is much lower than that in the existing simulator [36, 37]; the reason should be the facilitation of the guiding hand which can detect the rib before puncture. Blood vessel injury is much higher and without statistical significance because of the reconstruction model from the spiral CT which includes many blood capillaries; however, many of these vessel injuries can be ignored during the biopsy. The real-time respiratory motion effect during the VR biopsy simulator is demonstrated in Figure 12.

## 6. Conclusion

In this paper, a novel force modeling methodology for the biopsy therapy with respiratory motion using direct clinical data is proposed; the dynamic biopsy biomechanics experiment architecture is designed and constructed to facilitate the accurate modeling of the insertion force. In addition, clinical evaluation is conducted and performed, and VR-based training evaluation based on the proposed haptic rendering solution is also performed, which demonstrated the validity of the mechanical model and the feasibility of the evaluation. It is the first time to propose a dynamic force modeling and introduce the idea of continuations modeling of real-time biopsy force rendering. The principal contribution of this paper is to establish a complete system for the study of percutaneous biopsy training, including experimental construction, theoretical analysis, and surgical laboratory validation and evaluation, which is finally based on the VR training simulation assessment, which is a comprehensive research and verification process. The Fourier model was used to model the experimental data of different tissue types, which illustrated that the dynamic force modeling can effectively apply to several tissue types; it is further verified that the mechanical model proposed in this paper has a certain generalization. The future work will include the following: the experimental equipment needs to be optimized, such as the simulation of human respiratory system, and experiments need to be performed to enrich experimental data and types.

## Figures and Tables

**Table 1:** Parameters of tissue movement during respiration.

	Kidney (left)	Heart	Lung	Liver	Dorsal muscles
Motion (mm)	7.1 (4.5-9.8)	5.2 (2.4-7.9)	8.6 (5.2-12.0)	4.5 (3.0-6.0)	2.9 (0.3-5.5)
Breath cycle (s)	5 (4-6)				

**Table 2:** Renal biopsy components in the operation room and the virtual simulator.

Number	Operation room (Figure 4)	Virtual simulation (Figure 5)
1	Guide image recording	Original guide image
2	Audio recording	Hi-fi headphone
3	Ultrasound head	Ultrasound head
4	ATI Nano17	PHANTOM Omni
5	18G trocar needle	18G trocar needle
6	Patient	CT reconstruction
7	Force output	Force rendering
8	Surgical environment	HTC VIVE

**Table 3:** Fitting degree between the biomechanics data and our force model.

Phase	Fitting degree					
Type	1	2	3	4	5	Average
Kidney (left)	0.9975	0.9446	0.9939	0.9936	0.9980	**0.9855**
Liver	0.9996	0.9988	0.9998	0.9994	0.9999	**0.9995**
Lung	0.9997	1	1	0.9979	0.9993	**0.9994**
Heart	1	0.9999	1	0.9310	0.9999	**0.9862**
Dorsal muscles	0.9997	0.9979	0.9999	0.9999	0.9996	**0.9994**

**Table 4:** Fitting degree between clinical data and our force model.

Phase	1	2	3	4	5	Average
Fitting degree	0.9768	0.9953	0.9991	0.9980	0.9990	**0.9936**

**Table 5:** Results of GRS and objective evaluation on the VR-based training simulator.

	Experts (9)	Novice (18)	P value
GRS (range 1-5)
Identify anatomy	2.8 ± 0.1	3.2 ± 0.2	<0.001
Plan needle puncture	2.2 ± 0.2	3.1 ± 0.2	<0.001
Instrument use	2.1 ± 0.1	3.1 ± 0.1	<0.001
Ability to perform tasks	2.3 ± 0.1	3.4 ± 0.2	<0.001
Overall performance	2.5 ± 0.1	3.6 ± 0.2	<0.001
Objective assessment
Operation time (min)	6.8 ± 0.4	8.9 ± 0.5	<0.001
Fluoroscopy time (min)	6.2 ± 0.3	8.6 ± 0.3	<0.001
No. needle punctures	2.4 ± 0.2	3.8 ± 0.2	0.002
Infundibular injury	0.4 ± 0.08	1.2 ± 0.1	<0.001
PCS perforations	0.5 ± 0.1	1.8 ± 0.1	0.024
Rib injury	1.5 ± 0.2	2.7 ± 0.2	0.02
Blood vessel injury	2.1 ± 0.3	3.7 ± 0.3	0.07

**Table 6:** Results of subjective evaluation on the VR-based training simulator.

Subjective questionnaires	Expert (9)	Novice (18)
Dynamic force model effects (range 1-5)		
Realism of respiratory	3.7	3.9
Realism of haptic feedback	3.9	4.2
Realism of needle insertion	4.1	4.1
Haptic fatigue	2.3	1.4
System evaluation (range 1-5)		
VR graphic performance	3.9	4.2
Surgical tool manipulation	3.4	3.7
Visual fatigue	1.1	1.2
Comfortable HMC training	3.7	3.5
Hand-eye coordination training	4.1	4.5
Overall appraisal	**3.6**	**3.4**
Exceptions? (range 1-5)		
Surgical navigation	3.1	3.5
Surgical planning	4.2	4.7
Novice training	4.7	4.6
Resident practice	3.9	4.1
Surgical rehearsal	3.5	3.8

**Table 7:** Fitting results between existing fitting algorithms with experimental data (left kidney).

Phase	The fitting degree					
Model	1	2	3	4	5	Average
Polynomial	0.9934	0.6981	0.9573	0.8382	0.9463	0.8867
Nonlinear	0.9863	0.5190	0.9022	0.7791	0.7555	0.7880
Exponential	0.9770	0.5511	0.8644	0.6810	0.9028	0.7953
Our method	**0.9975**	**0.9446**	**0.9939**	**0.9936**	**0.9980**	**0.9855**

**Table 8:** Fitting results between existing fitting algorithms with clinical data (renal biopsy).

Phase	The fitting degree					
Model	1	2	3	4	5	Average
Polynomial	0.6248	0.8738	0.9390	0.9558	0.9980	0.8783
Nonlinear	0.6269	0.8420	0.9365	0.4726	0.7254	0.7207
Exponential	0.4076	0.8548	0.9375	0.3017	0.8331	0.6670
Our method	**0.9768**	**0.9953**	**0.9991**	**0.9980**	**0.9990**	**0.9936**

## Data Availability

The experimental data used to support the findings of this study are available from the corresponding author upon request.
